# Supplementary material for: Computational Study of Natural Compounds for the Clearance of Amyloid-Βeta: A Potential Therapeutic Management Strategy for Alzheimer’s Disease
Source: Molecules. 2019 Sep 5;24(18):3233. doi: 10.3390/molecules24183233 (PMC6767296; doi:10.3390/molecules24183233)
Supplement: Supplementary file 1 [file molecules-24-03233-s001.pdf]

molecules

Supplementary Materials

Computational Study of Natural Compounds for the Clearance of Amyloid-Beta: A Potential Therapeutic Management Strategy for Alzheimer's Disease

Syed Sayeed Ahmad [1], Haroon Khan [2,*], Syed Mohd. Danish Rizvi [3], Siddique Akber Ansari [4], Riaz Ullah [5], Luca Rastrelli [6], Hafiz Majid Mahmood [7] and Mohd. Haris Siddiqui [1,*]

1 Department of Bioengineering, Faculty of Engineering, Integral University, Lucknow, 226026, India
2 Department of Pharmacy, Abdul Wali Khan University Mardan 23200, Pakistan
3 Department of Pharmaceutics, College of Pharmacy, University of Hail, PO Box 2440, Ha'il – 81451, Saudi Arabia
4 Department of Pharmaceutical Chemistry, College of Pharmacy, King Saud University, P.O. Box: 2457, Riyadh, 11451, Saudi Arabia
5 Medicinal, Aromatic and Poisonous Plants Research Center (MAPRC), College of Pharmacy, King Saud University, PO box 2457, Riyadh 11451, Saudi Arabia
6 Dipartimento di Farmacia, University of Salerno, 84084 Fisciano, Italy
7 Department of Pharmacology, College of Pharmacy, King Saud University PO box 2457, Riyadh 11451, Saudi Arabia
* Correspondence: hkdr2006@gmail.com or haroonkhan@awkum.edu.pk (H.K.); mohdharis.siddiqui@gmail.com (M.H.S.); Tel.: +92-3329123171 (H.K.)

Table S1. Human Intestinal Absorption (HIA) value of compounds.

S.No.	Compound Name	HIA Value
**1.**	Vincamine	95.95%
**2.**	Ajmalicine	93.31%
**3.**	Emetine	96.59%
**4.**	Curcumin	94.40%
